# Evaluation of an AI Scribe Tool in the Emergency Department: A Single‐Arm Observational Study

**DOI:** 10.1111/1742-6723.70272

**Published:** 2026-05-06

**Authors:** Hamed Akhlaghi, Sam Freeman, Kevin Sun, Neo Nie, John Ding, Leo Chen, Eden Pham, Brendan Morrissey, Jonathan Karro

**Affiliations:** ^1^ Emergency Department St Vincent's Hospital Melbourne Victoria Australia; ^2^ School of Psychology, Faculty of Health Deakin University Melbourne Victoria Australia; ^3^ Department of Critical Care, Faculty of Medicine, Dentistry and Health Sciences University of Melbourne Melbourne Victoria Australia; ^4^ NexusMD.Ai Richmond Victoria Australia; ^5^ Australian Institute of Health Innovation, Macquarie University Sydney New South Wales Australia; ^6^ Department of Psychiatry The University of Melbourne Melbourne Victoria Australia; ^7^ Mental Health and Addiction Medicine St Vincent's Hospital Melbourne Victoria Australia

## Abstract

**Background:**

Generative artificial intelligence (AI) is reshaping the way clinicians record their clinical notes. AI‐scribe systems leverage generative AI capabilities to transcribe clinical encounters into draft clinical notes. In this study, we assessed clinician uptake and estimated modelled documentation time savings for an AI‐scribe system in an emergency department (ED).

**Method:**

ED physicians and trainees were provided access to an AI‐scribe for 5 weeks. Data from the first week were excluded. The transcript of each presentation, the initial AI‐generated clinical note and the final EMR clinical notes were used to calculate time to finalise AI‐assisted notes.

**Results:**

Forty ED consultants and 23 trainees accessed the AI‐scribe. Over the study period, nine consultants (22.5%) and 11 registrars (48%) used the system. The AI‐scribe was used for 248 ED presentations, including 185 (74.6%) by trainees and 63 (25.4%) by consultants. The system generated 44,489 words. Following clinician review, 18,140 words were added and 2274 were deleted prior to submitting the final clinical notes. For a clinician with an average typing speed, use of the AI‐scribe resulted in a time saving of 7.1 h of documentation. This was reduced to 4.9 h for rapid typer clinicians. Overall, the initial AI‐generated notes were modified on 1143 occasions. The most frequently revised section was the *history of presenting illness* (23.3%) followed by the *management plan* (22.9%).

**Conclusion:**

Uptake of AI‐scribe was higher among trainees than consultants, and the platform achieved substantial time savings. Future studies are required to quantify real‐time productivity gains over longer periods.

## 1 Introduction

Recent advances in generative artificial intelligence (AI) and natural language processing (NLP) have enabled new approaches to automating clinical documentation. Ambient or digital AI scribe systems combine automatic speech recognition with NLP to process clinician–patient conversations and draft encounter notes for clinician review. van Buchem et al. describe how these systems are designed to record the consultation, transcribe speech, extract medically relevant information and generate structured summaries that can be added to a patient's electronic medical record (EMR) [1]. These improvements in conversational AI and summarisation techniques allow AI scribes to create clinically accurate notes that can reduce documentation burden for clinicians. In ambient listening systems, audio from the consultation is transcribed and simultaneously followed by conversational AI processing and converted into a draft clinical note to be reviewed, edited (if required) and finalised by the clinician [2].

This hybrid automated–human workflow is designed to reduce the time clinicians spend typing or dictating, streamlining documentation. AI scribes are increasingly adopted to relieve administrative load, improve documentation efficiency and mitigate contributors to clinician workload and burnout [3, 4]. Emerging evidence positions AI scribes as a practical response to escalating documentation demands and the need for scalable, reliable strategies to reduce the burden of using EMRs [1]. Early evaluations of AI scribes highlight their potential to increase clinician engagement and decrease documentation time, despite current evidence remaining mixed and incomplete [2]. Current research on AI‐scribes has largely relied on clinicians' self‐reported perceptions of benefit rather than objective measures. Several AI‐scribe evaluations describe reductions in perceived documentation burden, improvements in usability, and greater job satisfaction, using surveys and subjective workload measures as primary endpoints [5]. In other studies, improvements in engagement and reduced cognitive load were similarly captured through self‐report, with only limited linkage to documentation activity within the EMR [6].

Emergency departments (EDs) operate in fast‐paced, high‐acuity environments characterised by frequent interruptions, rapid decision‐making and substantial documentation requirements. These conditions make the ED an ideal setting in which to evaluate the performance and clinical utility of AI scribes, particularly given the sustained administrative burden and escalation in documentation time identified across broader clinical settings [7]. Despite this, no published studies have examined AI scribe performance using objective data drawn directly from emergency medicine workflows. A recent scoping review found that most digital scribe studies focus on technical accuracy or limited usability testing, with very few assessing real‐world clinical impact or documentation efficiency [1]. Even among studies incorporating EMR‐derived data, the evidence remains concentrated in ambulatory and outpatient settings. For example, a peer‐matched cohort study of an ambient listening system demonstrated small but statistically significant reductions in EMR documentation time, yet results were limited to outpatient workflows and short follow‐up periods [2]. Other analyses using Epic Signal data similarly reported improvements in documentation time but these were confined to specialty or primary care clinics and did not include emergency medicine contexts [5]. To the authors' knowledge, there is no published study evaluating objective performance of AI scribes in EDs. This gap highlights the need for ED‐specific, quantitative evaluations capable of capturing real‐world efficacy and impact on ED documentation. The objectives of this study were to assess clinician uptake and estimate documentation time savings associated with the use of an AI scribe system in the ED.

## 2 Methods

This is a single‐arm observational study, conducted at St Vincent's Hospital Melbourne ED. All ED consultants and trainees working between 2 June 2025 and 6 July 2025 were provided access to the AI scribe software. An educational session outlining the procedures for using the AI scribe software was conducted for both consultants and trainees. The first week of the trial was designated as a pilot phase to familiarise the ED team with the software. A trained representative from the software vendor attended the ED during this initial week to assist with troubleshooting and to provide one‐on‐one training. Data from this week was excluded from the final analysis. Data collection commenced in the second week and continued until the last day of the fifth week (a total of 28 days). No personnel from the software vendor attended or contacted the ED during the data collection period. Using the AI scribe software was completely voluntary.

The study was reviewed and approved by the St Vincent's Hospital Melbourne Human Research Ethics Committee (HREC) (Project ID: 114749).

**FIGURE 1:**
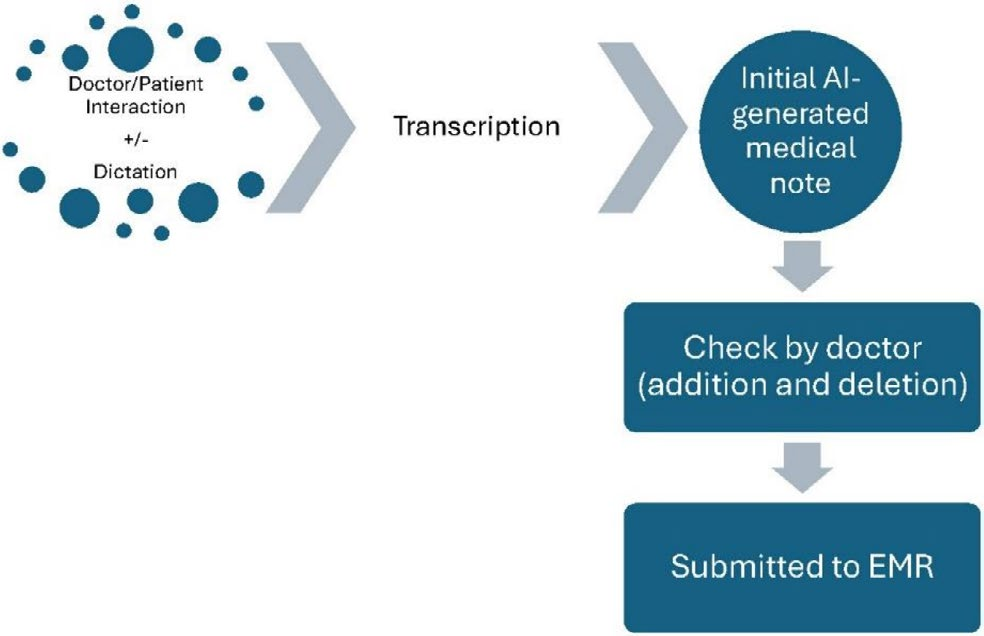
The schematic process of utilising AI‐scribe software to generate clinical notes ready for submission into the EMR.

For each encounter in this study, three levels of data were collected: (1) the main transcription of the medical consultation, (2) the AI‐generated notes with no correction by the clinician and (3) the clinical notes recorded in the patient's EMR after correction. The main transcriptions and AI‐generated notes were collected by the AI scribe software, whereas the final clinical notes were recorded in the hospital's EMR. The final clinical notes were manually extracted by the author (HA) from the EMR. Ten percent of cases were randomly selected for independent EMR data extraction by a second investigator (EP). Comparison of the two datasets demonstrated complete agreement, with no discrepancies identified. To be in compliance with ethical approval requirements, each note began with “***** AI‐Generated note for research approved by StV Ethics Committee *****” and ended with “***** End of AI‐generated note *****”. These markers were used to identify AI‐generated clinical notes recorded in the EMR (Figure 1).

The AI transcription could represent either an active capture of the real‐time interaction between the doctor and patient during the medical history taking consultation, a dictation of the clinical encounter by the doctor, or a mix of both, which included ambient listening of the history taking and dictating of physical examinations.

**FIGURE 2:**
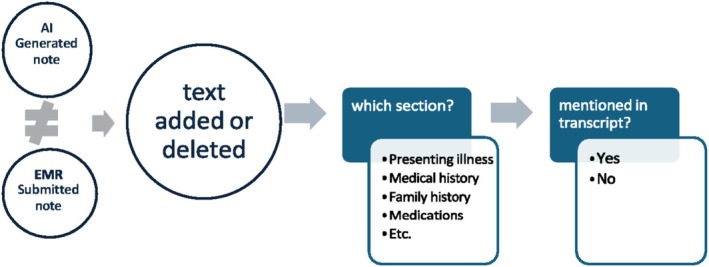
Process used to identify whether a text was added or deleted after the initial AI‐generated clinical note, and to determine which section of the clinical note it belonged. EMR: Electronic medical record.

The differences between the final AI‐generated notes submitted to the EMR to the initial AI‐generated notes and transcriptions were analysed to determine the extent of manual editions by the doctors. We also assessed what sections of the clinical note were altered and whether the added content by doctors was mentioned in the transcriptions (Figure 2). To achieve this, the final submitted EMR notes were compared with the initial AI‐generated notes to identify all additions and deletions. Each modification was then classified according to the relevant section of the final EMR note. In addition, the principle of large language modelling was applied to determine whether newly added content in the final EMR note was already present in the original transcript. This approach was necessary because added text may not match the verbatim transcript despite conveying the same clinical information. For further clarification, an example of the analytical output is provided in the Table S1.

To assess whether the AI scribe software reduces modelled documentation time, we calculated the time required for documentations as below:

1. Estimated time if the doctor manually typed the entire note. To do this, we calculated the total number of words in the final EMR note and estimated the time required for a doctor to type it manually, based on an average typing speed of 52 words per minute (WPM) for an average typist and 65 WPM for a fast typist [8, 9, 10].
2. Estimated time required when using the AI scribe:
  - Calculated the number of words in the AI‐generated initial note and the time required for the AI to generate them.
  - Estimated time for the doctor to read and review the AI‐generated note, based on a typical adult reading speed of 238 WPM [11, 12].
  - Determined the number of words added or deleted by the doctor and estimated the time required to perform these edits as above [10].
3. Total modelled documentation time using the AI scribe: The overall time required for an AI scribe note to be ready for EMR submission was calculated as the sum of (1) AI generation time, (2) doctor review (reading) time and (3) editing time.

## 3 Results

Forty ED consultants and 23 ED trainees were provided access to the AI scribe. By the completion of the study, the AI scribe was used by nine consultants (22.5%) and 11 trainees (48%). The AI scribe generated notes for 248 ED presentations: 185 (74.6%) produced by trainees and 63 (25.4%) by consultants. In total, the AI scribe software generated 44,589 words (median 168 words per each clinical note; IQR [133–219]).

After reviewing the initial AI‐generated notes, a total of 18,140 words were added (median = 50 words per each clinical note; IQR [12–113]) corresponding to 98,007 characters (median = 259 characters per each clinical note; IQR [61–639]), and deleted 2274 words (median = 0 words per each clinical note; IQR [0–6]) corresponding to a net character count of 13,656 (median = 0 character per each clinical note; IQR [0–49]).

The final EMR‐submitted notes contained a median of 203 words per entry (IQR [157–269]). A total of 54,140 words (327,577 characters) were entered over 248 separate entries. Based on an average typing speed of 52 WPM, this would require approximately 1041 min (17.4 h) to manually type the same volume of text. Using an assumed typing speed of 65 WPM for faster typers, the required time would decrease to 833 min (13.9 h).

The AI system generated each note in approximately 10 s, resulting in a total generation time of 2480 s (41 min or 0.7 h). Reading the 44,589 AI‐generated words for review and correction would take 3.1 h (average reading speed of 238 WPM). To add or delete the 20,414 words in total, doctors would require 393 min (6.5 h) at an average typing speed or 314 min (5.2 h) for faster typists. Therefore, it takes 10.3 h (0.7 + 3.1 + 6.5) for average typers or 9 h (0.7 + 3.1 + 5.2) for fast typers to complete AI‐assisted clinical notes.

**FIGURE 3:**
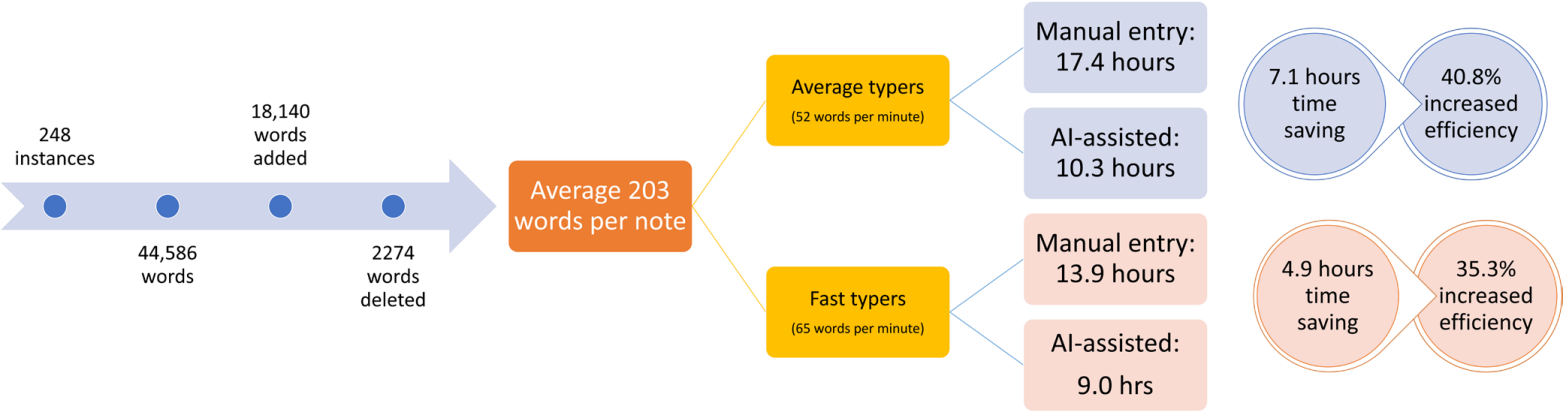
An infographic summary of the results.

Overall, compared with the estimated 17.4 h required for manual documentations considering all doctors typing at the average speed of 52 WPM, the use of AI‐assisted note generation reduced modelled documentation time by 7.1 h, equivalent to a 40.8% increase in efficiency. If all users were rapid typists (65 WPM), using the AI‐scribe saved 4.9 h, representing a 35.3% increase in efficiency (Figure 3).

Following generation of the initial AI‐generated clinical notes, a total of 1143 alterations were made by clinicians. Of these, 872 were additions (76.3%) and 271 were deletions (23.7%). The most frequently revised section was the *history of presenting illness* (266 revisions, 23.3%), followed by the *management plan* (262 revisions, 22.9%) and *impression* (191 revisions, 16.7%) (see Table 1 for more details).

**TABLE 1:** Detailed analysis of additional text or deleted sections after AI‐scribe generated the clinical note.

	Addition			Deletion	Total *N* (%)
	Was in transcript				
	Yes (%)	No (%)	Total		
HOPC^a^	158 (81)	37 (19)	195	71	266 (23.3)
Management Plan	148 (76.7)	45 (23.3)	193	69	262 (22.9)
Impression	147 (95.5)	7 (4.5)	154	37	191 (16.7)
Pmx^b^	93 (83)	19 (17)	112	35	147 (12.9)
Social History	90 (79.6)	23 (20.4)	113	30	143 (12.5)
Medications	86 (81.9)	19 (18.1)	105	29	134 (11.7)

^a^ HOPC: History of presenting complaint.

^b^ Pmx: PAST medical history.

## 4 Discussion

This study provides, to the best of our knowledge, the first assessment of AI scribe use within an Australian public hospital ED and suggests that efficiency gains of 35.3%–40.8% are achievable in a high‐pressure clinical environment based on the modelled documentations. This clinically relevant and meaningful reduction in modelled documentation time, even when conservative assumptions were applied to reading and typing speeds, assisted the workload required to complete EMR‐submitted notes. These findings strengthen the evidence base by moving beyond self‐reported perceptions of workload reduction; instead, using direct, objective measurements from transcripts, AI‐generated notes and final EMR entries. Overall, the study shows that AI scribe technology can be successfully integrated into ED workflows and can produce quantifiable time savings that translate into increased documentation efficiency. The findings of this study align with and extend the existing body of research on AI scribe technology and its clinical integration. Prior evaluations of AI scribes have typically reported reductions in perceived documentation burden, improved usability and greater clinician satisfaction. However, they have typically relied on self‐reported measures rather than direct observation or routinely collected EMR data [5]. Studies examining physician burnout also show that clinicians often describe AI scribes as reducing mental workload by supporting more efficient documentation; yet these benefits have been captured through surveys rather than objective performance data [6].

Only a small number of published studies have used platform‐generated or EMR‐derived measures to quantify changes in documentation time. For example, a controlled cohort study of an ambient listening system found statistically significant improvements in documentation efficiency but noted that these effects were small and mainly observed in lower‐acuity outpatient settings [2]. A recent scoping review of digital scribes showed that most research has focused on technical model performance or limited usability testing, with very few studies assessing clinical utility or real‐world workflow impact [1]. The present study contributes new evidence by providing an ED‐specific, objective assessment on AI‐scribe uptake and documentation efficiency. While the magnitude of time saved was broadly consistent with reductions reported in outpatient studies, this is the first evaluation to demonstrate similar gains in a complex ED environment. Although this study demonstrated that the use of an AI scribe system can save time in the ED, its effectiveness and efficiency may vary at the individual clinician level. Some doctors may perceive that using an AI scribe for documentation increases the time and workload required to complete clinical notes. Further studies are therefore needed to evaluate the impact of AI scribe systems at the individual level.

The study also extends existing evidence by analysing the content and distribution of clinician edits across note sections, providing a more detailed understanding of where generative models perform well and where improvement is most needed. The pattern of edits observed in this study offers insight into how generative AI models function within emergency medicine workflows. The AI scribe performed well in structured or semi‐structured sections such as past history and medications but required more clinician input in narrative components such as the history of presenting illness, management plan and impression. This reflects the limitations identified in previous evaluations of digital scribe technologies, where models may not accurately or naturally capture the nuance, chronology and contextual detail present in complex clinical narratives [1]. In the ED setting, this suggests that while AI scribes can reliably support routine documentation tasks, clinician oversight remains essential for sections requiring interpretation or clinical reasoning.

We found different uptake between trainees and consultants, with uptake more readily exhibited by trainees. This is consistent with emerging adoption patterns with other digital innovations in which early‐career clinicians appear to be earlier adopters of new technologies into their workflow [5]. Understanding the factors that shape adoption within the ED will be important for implementation planning, particularly given the need for systems that complement rather than disrupt existing clinical processes.

A strength of this study is its reliance on objective, encounter‐level data generated within a naturalistic, high‐volume ED setting. Unlike previous AI scribe evaluations, which have typically relied on clinician self‐report perceptions [5, 6], this study analysed the complete documentation pathway, including transcripts, AI‐generated drafts and EMR‐recorded notes. Additionally, our method enabled evaluation of content revisions to the AI scribe notes, which have received limited evaluation in prior studies. This granular analysis of AI scribes in clinical settings clarified when generative models perform reliably and when clinician correction remains necessary. These insights are essential for future system refinement and support the call for more sophisticated evaluations of AI scribe performance and workflow integration [1].

Another strength of the study is that it was conducted in a complex, high‐acuity environment where documentation demands are substantial. EDs are known to present unique challenges for digital tools due to their pace, variability and interruption‐heavy workflows. Demonstrating meaningful efficiency gains in this context adds important evidence to a research area that has often previously been limited to outpatient settings [13]. Finally, by capturing real‐world uptake patterns, the study provides practical insight into how different clinician groups engage with AI scribes. This contributes to a more realistic understanding of implementation dynamics, which is critical for scaling digital documentation solutions across diverse clinical teams.

However, the results of this study should be interpreted with consideration of its limitations. First, time‐saving estimates were derived from modelled documentation time rather than direct observation, which may over or underestimate true performance. In addition, AI‐generated notes may differ in length and verbosity from traditionally written notes, and this may have influenced the time saving observed in this study. The speed of reading and writing was derived from the general population rather than ED clinicians. However, this approach provides reasonable estimates to facilitate quantitative efficiency calculations and is a common method in digital scribe research where time‐and‐motion methods are difficult to apply in busy clinical environments [1]. Additionally, the usage of the AI scribe platform was voluntary, and ED hospital medical officers and interns did not take part in this study, which can contribute to less usage of the AI tool during this study. Second, we did not compare the actual time spent by clinicians completing a documentation task with and without an AI scribe software for the same clinical task. Doing so would have improved comparison validity but was challenging to implement in a busy, real‐world ED environment due to the extra time required to complete both tasks [14]. Third, the automated approach to identifying additions and deletions may introduce minor inaccuracies in quantifying edits. Finally, this was a single‐site evaluation within one Australian ED. Given known variability in documentation practises across services, findings may not be fully generalisable to other EDs or health systems. A multi‐site study and a broader cross‐section of the Australian population is required to better understand the impact of AI scribe systems on EDs.

## 5 Conclusion

The findings of this study suggest that AI scribes have value in the ED, where high documentation volumes and frequent interruptions contribute to workload pressures. Even with conservative assumptions, the AI scribe produced meaningful reductions in the time required to generate complete clinical notes based on the modelled documentation, indicating that this technology may be well suited to supporting documentation in fast‐paced environments.

## Author Contributions

Each author certifies that their contribution to this work meets the standards of the International Committee of Medical Journal Editors.

## Funding

The authors have nothing to report.

## Disclosure

The NexusMD.ai company (www.nexusmd.ai) provided the AI‐scribe software used in this study. The software is specifically designed for use in EDs and complies with the Australian AI regulatory and ethical framework.

## Ethics Statement

The ethics approval for this work was granted by St Vincent's HREC (Human Research Ethics Committee) with an approval number of LRR 011/25. All the authors hold a valid Certificate of Good Clinical Practice (GCP). All data were de‐identified before analysis to ensure participant privacy, and the study adhered to the principles of the Declaration of Helsinki.

## Conflicts of Interest

A/Prof Hamed Akhlaghi serves as a medical advisor to NexusMD.ai. This study was conducted independently, with no funding, input or influence from NexusMD.ai on the study design, data analysis or reporting of results. Mr. Kevin Sun is Chief Technology Officer of NexusMD.ai. Mr. Neo Nie is Chief Product Officer of NexusMD.ai. Dr. John Ding is a medical director of NexusMD.ai.

## Supporting information


**Table S1:** An example of the output to identify additions and deletions of the final EMR note and the relevant sections. Italic bold words are the additions and underlined bold words are deletions.

## Data Availability

The data that support the findings of this study are available on request from the corresponding author. The data are not publicly available due to privacy or ethical restrictions.

## References

[1] M. M. van Buchem , H. Boosman , M. P. Bauer , I. M. J. Kant , S. A. Cammel , and E. W. Steyerberg , “The Digital Scribe in Clinical Practice: A Scoping Review and Research Agenda,” npj Digital Medicine 4, no. 1 (2021): 57. 33772070 10.1038/s41746-021-00432-5 PMC7997964

[2] T. Haberle , C. Cleveland , G. L. Snow , et al., “The Impact of Nuance DAX Ambient Listening AI Documentation: A Cohort Study,” Journal of the American Medical Informatics Association 31, no. 4 (2024): 975–979. 38345343 10.1093/jamia/ocae022 PMC10990544

[3] T. I. Leung , A. J. Coristine , and A. Benis , “AI Scribes in Health Care: Balancing Transformative Potential With Responsible Integration,” JMIR Medical Informatics 13 (2025): e80898. 40749188 10.2196/80898 PMC12316405

[4] A. L. Terry , K. Thompson , S. Tsuei , and D. J. Lizotte , “Stepwise Considerations When Using Artificial Intelligence Tools for Administrative Tasks in Primary Care,” Canadian Family Physician 71, no. 6 (2025): e90–e93. 40523749 10.46747/cfp.7106e90 PMC12264532

[5] M. J. Duggan , J. Gervase , A. Schoenbaum , et al., “Clinician Experiences With Ambient Scribe Technology to Assist With Documentation Burden and Efficiency,” JAMA Network Open 8, no. 2 (2025): e2460637. 39969880 10.1001/jamanetworkopen.2024.60637 PMC11840636

[6] S. J. Shah , A. Devon‐Sand , S. P. Ma , et al., “Ambient Artificial Intelligence Scribes: Physician Burnout and Perspectives on Usability and Documentation Burden,” Journal of the American Medical Informatics Association 32, no. 2 (2025): 375–380. 39657021 10.1093/jamia/ocae295 PMC11756571

[7] S. Agius , C. Magri , and V. Cassar , “A Cognitive Task Analysis for Developing a Clinical Decision Support System for Emergency Triage,” Journal of Emergency Nursing 51, no. 6 (2025): 1028–1045. 40626939 10.1016/j.jen.2025.05.013

[8] D. Talbot , “Typing Speed Statistics,” (2025), WordsRated.

[9] Typing.com , “The Time Savings of Fast Typists,” (2025), Typing.com Articles.

[10] V. Dhakal , “Observations on Typing From 136 Million Keystrokes,” in Proceedings of the 2018 CHI Conference on Human Factors in Computing Systems (Association for Computing Machinery, 2018), 646.

[11] K. Rayner , B. R. Foorman , C. A. Perfetti , D. Pesetsky , and M. S. Seidenberg , “How Psychological Science Informs the Teaching of Reading,” Psychological Science in the Public Interest 2, no. 2 (2001): 31–74. 10.1111/1529-1006.00004. 11878018

[12] M. Brysbaert , “How Many Words Do We Read Per Minute? A Review and Meta‐Analysis of Reading Rate,” Journal of Memory and Language 109 (2019): 104047.

[13] M. M. van Buchem , I. M. J. Kant , L. King , J. Kazmaier , E. W. Steyerberg , and M. P. Bauer , “Impact of a Digital Scribe System on Clinical Documentation Time and Quality: Usability Study,” Jmir ai 3 (2024): e60020. 39312397 10.2196/60020 PMC11459111

[14] Y. Guo , D. Hu , J. Wang , et al., “Ambient Listening in Clinical Practice: Evaluating EPIC Signal Data Before and After Implementation and Its Impact on Physician Workload,” Studies in Health Technology and Informatics 329 (2025): 653–657. 40775939 10.3233/SHTI250921 PMC13039322

