# Supplementary material for: Evaluation of an AI Scribe Tool in the Emergency Department: A Single‐Arm Observational Study
Source: Emerg Med Australas. 2026 May 6;38:e70272. doi: 10.1111/1742-6723.70272 (PMC13147221; doi:10.1111/1742-6723.70272)
Supplement: Supplementary file 1 — Table S1: An example of the output to identify additions and deletions of the final EMR note and the relevant sections. Italic bold words are the additions and underlined bold words are deletions. [file EMM-38-0-s001.docx]

**Supplementary Table 1:**

An example of the output to identify additions and deletions of the final EMR note and the relevant sections. Italic bold words are the additions and underlined bold words are deletions.

| **Section PC:**  **Added:** 45yo  **Found in trans:** Yes  **Proof:** A 45-year-old male brought in by ambulance for several episodes of haematemesis on background of heavy alcohol intake.  **Section HOPC:**  **Deleted:** jaundice, Family convinced to call ambulance  **Found in trans:** Yes  **Proof:** Niece has noticed that over the past three months, his gums have started to bleed, he's become progressively jaundiced and abdomen has become more swollen and distended. ... However, family convinced him to call an ambulance today.  **Section O/E:**  **Added:** Compliant with exam, cannula No hepatic flap Initial gas: Hb 114, Lct 7.4  **Found in trans:** No  **Section Impression:**  **Added:** haematemesis, jaundice, ascites likely coagulopathy ?underlying varices  **Found in trans:** Yes  **Proof:** A 45-year-old male brought in by ambulance for several episodes of haematemesis on background of heavy alcohol intake... he's become progressively jaundiced and abdomen has become more swollen and distended... Abdomen is descended with what appears to be a moderate volume ascites, shifting dullness present... Impression, likely first presentation of alcoholic cirrhosis with haematemesis and bleeding gums.  **Deleted:** haematemesis bleeding gums  **Found in trans:** Yes  **Proof:** A 45-year-old male brought in by ambulance for several episodes of haematemesis on background of heavy alcohol intake... Niece has noticed that over the past three months, his gums have started to bleed...  **Section Management Plan:**  **Added:** infusion thereafter 8mg/hr Vitamin K if INR >1.2 consideration of urgent  **Found in trans:** No | ***A 45-year-old male brought in by ambulance for several episodes of haematemesis on background of heavy alcohol intake.*** Patient has been drinking approximately 1 to 2 bottles of red wine daily over past 18 months following deterioration of mental health. Niece has noticed that over the past three months, his gums have started to bleed, he's become progressively jaundiced and abdomen has become more swollen and distended. Over past 48 hours, patient has had several episodes of haematemesis, unfortunately unable to quantify volume or colour of vomitus. patient previously not. Unfortunately, unable to quantify volume or colour of vomitus. Patient previously not amenable to coming to hospital. However, family convinced him to call an ambulance today. On arrival, found to be a GCS of 14. jaundice, smelling strongly of alcohol, with blood around the mouth. Tachycardic at 120 otherwise vital stable afebrile. No past medical history of note, no drug history, no known drug allergies. Patient lives at home with his wife and Six-month-old child. He is a non-smoker. Alcohol history as previously described, no illicit drug use. On examination, strong smell of alcohol, dried Alcohol history as previously described, no illicit drug use. On examination, strong smell of alcohol, dried black vomitus around mouth and coating tongue. Airways patents, sats of 96% on room air, rests of 20. Chest clear, bilaterally no wheeze. Very cold peripheries with sluggish central cap refill of 4 seconds, dry tongue, heart sounds dual, no murmur, heart rate 120 to 125 on monitor, blood pressure, 145 on 66. GCS of 14, eyes 4, verbal 4, motor 6, afebrile. GCS of 14, eyes 4, verbal 4, motor 6, afebrile 36-6. Abdomen is descended with what appears to be a moderate volume ascites, shifting dullness present. Calves are soft, non-tender, no evidence of petechiae or bleeding elsewhere. Niece at bedside confirms no history of falls. Impression, likely first presentation of alcoholic cirrhosis with haematemesis and bleeding gums. Plan bloods, including group and holds, ECG, PPI cover, high dose. plan, bloods including group and holds, ECG, PPI cover, high dose, AWS, IV thiamine high dose, fluids, ultrasound, Abdo to look for cirrhosis, chest x-ray to rule out, consolidation, Gastro admission with you to scope. | ***** AI-Generated note for research approved by StV Ethics Committee ***** Male presenting with haematemesis HOPC: Several episodes of haematemesis over the past 48 hours Heavy alcohol intake, 1 to 2 bottles of red wine daily for 18 months Bleeding gums, progressive jaundice, and swollen abdomen over the past three months GCS of 14 on arrival, jaundice, blood around mouth Tachycardic at 120, afebrile **Family convinced to call ambulance** PMH: None Meds: None Allergies: NKDA SH: Lives at home with wife and six-month-old child Non-smoker No illicit drug use O/E: Strong smell of alcohol Dried black vomitus around mouth and tongue Airways patent, sats 96% on room air, resps 20 Chest clear, no wheeze Cold peripheries, sluggish capillary refill 4 seconds, dry tongue Heart sounds dual, no murmur, HR 120-125, BP 145/66 GCS 14, afebrile 36.6 Abdomen distended with moderate ascites, shifting dullness Calves soft, non-tender, no petechiae or bleeding elsewhere Impression: Likely first presentation of alcoholic cirrhosis with **haematemesis and bleeding gums** Management Plan: Bloods including group and hold ECG PPI **cover,** high dose AWS IV thiamine high dose Fluids Abdominal ultrasound to assess for cirrhosis Chest x-ray to rule out consolidation Gastroenterology admission for endoscopy ***** End of AI-generated note ***** | ***** AI-Generated note for research approved by StV Ethics Committee ********45yo*** Male presenting with haematemesis HOPC: Several episodes of haematemesis over the past 48 hours Reported by wife, who had told niece 'there is blood everywhere' Niece unable to quantify volume otherwise, or colour of vomitus Heavy alcohol intake, 1 to 2 bottles of red wine daily for 18 months Bleeding gums, progressive jaundice, and swollen abdomen over the past three months Patient has avoided hospital/seeing GP since onset of heavy ETOH Family convinced him to comply with AV today GCS of 14 on AV arrival, jaundiced, blood around mouth Tachycardic at 120, afebrile No vomits witnessed by AV PMH: None Meds: None Allergies: NKDA SH: Lives at home with wife and six-month-old child Non-smoker No illicit drug use O/E: Compliant with exam, cannula Strong smell of alcohol Dried black vomitus around mouth and tongue Airways patent, sats 96% on room air, resps 20 Chest clear, no wheeze Cold peripheries, sluggish capillary refill 4 seconds, dry tongue Heart sounds dual, no murmur, HR 120-125, BP 145/66 GCS 14, afebrile 36.6 No hepatic flap Abdomen distended with moderate ascites, shifting dullness Calves soft, non-tender, no petechiae or bleeding elsewhere Initial gas: Hb 114, Lct 7.4 Impression: Likely first presentation of alcoholic cirrhosis with ***haematemesis, jaundice, ascites and likely coagulopathy ?underlying varices*** Management Plan: Bloods including group and hold ECG PPI cover high dose bolus + infusion thereafter 8mg/hr AWS IV thiamine high dose Fluids Vitamin K if INR >1.2 Abdominal ultrasound to assess for cirrhosis Chest x-ray to rule out consolidation Gastroenterology admission for consideration of urgent endoscopy ***** End of AI-generated note ***** |
| --- | --- | --- | --- |
